# Validation and Psychometric Properties of the French Versions of the Environmental Reward Observation Scale and of the Reward Probability Index

**DOI:** 10.5334/pb.bg

**Published:** 2015-06-10

**Authors:** Aurélie Wagener, Sylvie Blairy

**Affiliations:** 1 Behavioural and Cognitive Clinical Psychology Unit, University of Liège, Liège, Belgium

**Keywords:** environmental reward, Environmental Reward Observation Scale, Reward Probability Index, validation, psychometric properties

## Abstract

**Background:** Low levels of environmental rewards have been related to depression on a number of occasions in the scientific literature. Two scales have been created to assess environmental rewards: the Environmental Reward Observation Scale (EROS) and the Reward Probability Index (RPI). This study aims to validate the French versions of these two scales. **Method:** 466 non-clinical adults completed an online survey assessing environmental rewards, depression, anxiety, activation, avoidance and behavioural systems. Confirmatory factor analyses were performed to assess the factorial structures of the French EROS and RPI. **Results:** A one-factor solution for the EROS and a two-factor solution for the RPI best fitted the data. High levels of internal consistency were found for both the EROS and the RPI. Convergent validity was also examined, revealing that high environmental rewards appear to be related to activation and behavioural activation system. **Conclusion:** The French versions of the EROS and the RPI appear to be reliable assessments of environmental rewards.

## Introduction

In the past two decades, there has been a revival of interest for behavioural treatments of depression. Lejuez, Hopko and Hopko (2001) and Lejuez, Hopko, Acierno, Daughters and Pagoto (2011) on the one hand, and Martell, Addis and Jacobson (2001) and Martell, Dimidjian and Herman-Dunn (2013) on the other hand, have developed paralleled and revised versions of the behavioural treatment of depression called Behavioural Activation. These psychotherapeutic approaches are based on the behavioural theory of depression, and more specifically on the principles of operant conditioning (Lewinsohn, 1974). These psychotherapeutic strategies aim to increase one’s contacts with positive reinforcements in his/her environment. Alongside this revival of interest for behavioural treatments of depression, there has also been an increased interest in the notion of environmental rewards, which is fundamental in the behavioural approach of depression (Carvalho & Hopko, 2011; Correia, Carey & Borsari, 2002; Hopko, Armento, Cantu, Chambers & Lejuez, 2003). Environmental rewards can be conceptualised as the perception of the positive or negative value of environmental experiences and activities available in one’s environment (Armento & Hopko, 2007). These experiences and activities can occur in several areas of life such as relationships (e.g., having a pleasant conversation with a friend) and work (e.g., receiving a promotion). It is noteworthy that the rewarding value of a particular event depends on personal values and therefore may differ across individuals. For instance, for a recreational activity, Miss A may enjoy taking walks and observing nature, while Miss B may prefer to visit a shop.

The behavioural theory of depression conceptualises a decrease in access to and in the frequency of environmental rewards as causal factors predicting the beginning and the maintenance of clinical depression (Carvalho, Trent & Hopko, 2011; Hopko, Lejuez, Ruggiero & Eifert, 2003; Lewinsohn, 1974). Other factors include the reinforcement of depressive behaviours and the punishment of healthy ones (Armento & Hopko, 2007; Carvalho, Gawrysiak et al., 2011; Lewinsohn, Sullivan & Grosscup, 1980). More precisely, according to Carvalho, Gawrysiak et al. (2011), a low level of response-contingent positive reinforcement (RCPR) is one of the critical predictors of clinical depression. The impact and the characteristics (frequency, certainty and magnitude) of environmental rewards have also been investigated (Armento & Hopko, 2007). Indeed, Hopko, Lejuez et al. (2003) highlighted that depressed individuals rated themselves as more passive than healthy controls. Moreover, depressed individuals spent more time in behaviours leading to minimal pleasure and less time in behaviours leading to extreme pleasure than healthy controls. Finally, Hopko, Lejuez et al. (2003) demonstrated that depressed individuals engaged significantly more often in behaviours which are perceived as unlikely to result in future rewards than healthy controls.

A RCPR is the process by which the occurrence of some behaviours increases due to the presence of a rewarding consequence (Carvalho, Trent et al., 2011). For example, a student will keep on studying if he obtains positive results or, a shy person may attend to more social events if he/she experienced a positive first outing. Therefore, when individuals lose contact with positive reinforcement, they might experience sad feelings and gradually become depressed (Kanter, Busch & Rusch, 2009). Lewinsohn et al. (1980) conceptualised the decrease in RCPR as the consequence of the combination of the following four variables: (a) a decrease in the number of reinforcing events (number’s decrease), (b) a decrease in the availability of these reinforcers in the environment (availability’s decrease), (c) an inability to experience rewarding contingencies due to inadequate instrumental behaviours (inability) and (d) an increase in the exposure to aversive and unpleasant environmental experiences (aversive exposure) (MacPhillamy & Lewinsohn, 1974). Psychotherapeutic strategies such as behavioural activation treatments aim to counteract the decrease in RCPR by increasing behaviours which allow contacts with pleasant events (positive reinforcement) as well as by decreasing aversive events (Lewinsohn et al., 1980; Manos, Kanter & Busch, 2010). This will allow more contacts with RCPR and then improve the affective state (Cuijpers, van Straten & Warmerdam, 2007; Manos et al., 2010; Mazzucchelli, Kane & Rees, 2010). Therefore, parsimonious and psychometrically sound assessment tools to measure the relationships between mood, behaviours and RCPR would be of interest for both clinical practice and research (Armento & Hokpo, 2007; Manos et al., 2010). Nonetheless, assessing RCPR directly is exceedingly complicated because it would require a relevant measure of behaviour, control over the subject’s environment and long periods of observation (MacPhillamy & Lewinsohn, 1974; Manos et al., 2010). Consequently, researchers who have aimed to develop measures of RCPR have focused on measuring environmental rewards, considered as indirect indicators of RCPR. Such measurement tools might help clinicians to assess the extent to which psychotherapeutic interventions actually modify client’s perception of environmental rewards. Moreover, as environmental rewards appear to influence the aetiology of several psychopathologies (Armento & Hopko, 2007), these tools could be employed in the study of a great variety of mental disorders (e.g., bipolar disorders, substance abuse disorders). These arguments have encouraged Armento and Hopko (2007) to develop the Environmental Reward Observation Scale, and Carvalho, Gawrysiak et al. (2011) to develop the Reward Probability Index. Because no assessment tools of environmental rewards are currently available in French, these arguments also encourage us to translate and validate these scales in French which is the aim of the current study. It appears that Armento and Hopko (2007) and Carvalho, Gawrysiak et al. (2011) used the term “reward” rather than “reinforcement” based on the neurobiological literature examining brain reward systems (Manos et al., 2010). According to White (1989), these two terms are quite different. Indeed, White (1989) wrote that “*reward refers to the fact that certain environmental stimuli have the property of eliciting approach responses. […] Reinforcement refers to the tendency of certain stimuli to strengthen learned stimulus-response tendencies*.” Nevertheless, these scales aim to assess the above-mentioned aspects of RCPR.

### The Environmental Reward Observation Scale

Armento and Hopko (2007) developed the Environmental Reward Observation Scale (EROS) to be a proxy measure of RCPR by assessing the subjective experience of reinforcement. Items were designed to measure increased behaviours and positive affects as consequences of rewarding environmental experiences during the last months (e.g., “*In general, I am very satisfied with the way I spend my time*”, “*The activities I engage in usually have positive consequences*”). Armento and Hopko (2007) administrated their scale to a sample composed of 202 undergraduate students which mean age was 19.6 years (*SD* = 2.7) (69.80% women). An exploratory factor analysis underlined a one-factor solution as being the best fit, accounting for 43% of the variance. These results were corroborated by a confirmatory factor analysis. The EROS one-factor structure demonstrated good internal consistency since Cronbach alpha was 0.85 (Tavakol & Dennick, 2011) and good convergent validity since EROS showed moderate-to-strong correlations with measures of depression and with measures of the behavioural activation system. Finally, test-retest reliability of EROS was excellent (Armento & Hopko, 2007).

### The Reward Probability Index

Carvalho, Gawrysiak et al. (2011) developed the Reward Probability Index (RPI) to evaluate the magnitude of the environmental rewards during the last months and, more precisely, the probability of experiencing rewards. They assessed the factorial structure of their scale in a non-clinical sample composed of 269 undergraduate students (66.9% women; mean age = 19.6, *SD* = 3.5). Confirmatory factor analyses highlighted that a two-factor solution resulted in a better fit compared to the expected four-factor structure (corresponding to the four aspects of RCPR: (a) number’s decrease, (b) availability’s decrease, (c) inability, (d) aversive exposure) and compared to a higher-order unifactorial model (Carvalho, Gawrysiak et al., 2011). Therefore, factor 1, called “*Reward Probability*” (11 items), combined items of “*Potentially reinforcing events*” and “*Instrumental behaviours*” (e.g., “*I have many interests that bring me pleasure*”, “*I have the abilities to obtain pleasure in my life*”). Factor 2, called “*Environmental Suppressors*” (9 items), combined items of “*Availability of reinforcement*” and “*Aversive and unpleasant experiences*” (e.g., “*I have few financial resources, which limits what I can do*”, “*It seems like bad things always happen to me*”). This two-factor structure explained 41.1% of the variance. This RPI two-factor structure demonstrated good internal consistency since Cronbach’s alpha for the entire scale was 0.88 (for factor 1, α = 0.80 and for factor 2, α = 0.87) and good convergent validity since RPI was moderately-to-strongly correlated with scores of depression and measures of activity and avoidance. Finally, test-retest reliability of RPI was excellent (r = 0.83 for factor 1 and r = 0.86 for factor 2) (Carvalho, Gawrysiak et al., 2011).

No assessment tools of environmental rewards are currently available in French. Thus, the main aim of the present study was to validate French translations of the Environmental Reward Observation Scale and the Reward Probability Index. More precisely, we translated the two scales, and examined their factor structures, reliabilities and convergent validities. Confirmatory factor analyses assessed the original one-factor structure of the EROS and the two-factor structure of the RPI. Because the original factorial structures were adequate, the goodness-of-fit indices of our confirmatory factor analyses were also expected to be adequate. The EROS and the RPI were expected to correlate positively with measures of activation and the behavioural activation system and to correlate negatively with measures of depression, anxiety, avoidance and the behavioural inhibition system.

## Method

### Participants and procedure

The current sample comprised 466 non-clinical adults (119 men; 347 women) with an average age of 36.5 years (range = 18–69, *SD* = 14.13). Six percent of the sample had a level of education lower than the secondary, 31% a secondary education one and 63% had a high level of education. Regarding job status, 49% of the participants were in full time employment, 22% were students, 9% retired, 8% unemployed, 5% were persons on sick-leave and 7% “unknown” (these participants did not specify their job status).

Participants were recruited via email, through personal contacts and by announcements on social networks (the study’s aim was described and a link to the survey was provided). Participants completed the online survey anonymously. The survey included questions concerning personal information (socio-demographic data), the French versions of the EROS and the RPI and four other questionnaires: the Beck Depression Inventory II (Beck, Steer & Brown, 1996; Centre de Psychologie Appliquée, 1996), the State-Trait Anxiety Inventory – Form B (Gauthier & Bouchard, 1993; Spielberger, Gorsuch, Lushene, Vagg & Jacobs, 1983), the Behavioural Activation for Depression Scale – Short Form (Manos, Kanter & Luo, 2011; Wagener, Van der Linden & Blairy, 2015), and the Behavioural Inhibition System/Behavioural Activation System Scale (Caci, Deschaux & Balé, 2007; Carver & White, 1994). The administration of these scales was part of another study conducted by the authors of the present paper. The study was approved by the local ethics committee.

### Instruments

#### The Environmental Reward Observation Scale (EROS) and the Reward Probability Index (RPI)

The EROS assesses the subjective experience of reinforcement. This scale is a Likert scale composed of ten 4-point items (varying from 1 = *Strongly disagree* to 4 = *Strongly agree*). Scores range from 10 to 40, with higher scores indicating more subjective experiences of environmental rewards (items 2, 5, 6, 7 and 9 are reverse scored) (Armento & Hopko, 2007). The RPI assesses reward probability. The RPI is a Likert scale consisting of twenty 4-point items (varying from 1 = *Strongly disagree* to 4 = *Strongly agree*). Scores range from 20 to 80, and higher scores indicate more reward probability and less environmental suppressors (Carvalho, Gawrysiak et al., 2011). Descriptive analyses of these scales are presented in the results section.

The EROS and the RPI were first translated into French by the authors and then back-translated into English by a bilingual expert. Discrepancies between the original and translated versions were discussed until that the translation seemed appropriate. Thirty-five bilingual adults completed both language versions of these two scales with an inter-test period of a few days (the order of the languages was counterbalanced: 18 French-English, 17 English-French). The order of the items in the French version was different from the order of the items in the English version. The EROS and the RPI’s translations seemed internally consistent as Cronbach’s alphas for the entire scales were respectively 0.90 and 0.93^1^ (Tavakol & Dennick, 2011). Correlations between the scores obtained by these bilingual adults on both language versions of the EROS and the RPI were computed (Table 1). These correlations indicate strong positive relationships between the scores on the original and translated versions, which seems to indicate an adequate match between these two versions.

**Table 1:** Pearson’s correlations between the original and translated versions of the EROS and of the RPI^1^ (*n* = 35).

Translated version	Original versions		

	**EROS**	**RPI – Factor 1**	**RPI – Factor 2**

EROS	0.89^**^	–	–
RPI – Factor 1	–	0.87^**^	–
RPI – Factor 2	–	–	0.82^**^

^1^^*^ p < 0.05, ^**^ p < 0.001

#### Beck Depression Inventory – II (BDI-II)

The BDI-II assesses the presence and severity of depressive symptoms that have occurred in the last two weeks according to DSM-IV criteria (Beck et al., 1996). The BDI-II is a Likert scale composed of twenty-one 4-point items (from 0 to 3) with scores ranging from 0 to 63 (e.g., “*I’m so sad or unhappy that I can’t stand it*”). The total score is equal to the sum of all of the items, with higher scores indicating more depressive symptoms. The French version of the BDI-II was used (Centre de Psychologie Appliquée, 1996).

#### State-Trait Anxiety Inventory – Form B (STAI-B)

The STAI assesses state and trait anxiety (Spielberger et al., 1983). In this study, only trait anxiety was evaluated (Form B). The STAI-B is a Likert scale composed of twenty 4-points items (from 1 = *Almost never* to 4 = *Almost always*) (e.g., “*I worry too much over something that really doesn’t matter*”). The total score is equal to the sum of all item scores, with higher scores indicating higher trait-anxiety. The French version of the STAI- B was used (Gauthier & Bouchard, 1993).

#### Behavioural Activation for Depression Scale – Short Form (BADS-SF)

The BADS-SF assesses the level of behavioural activation of the last week (Manos et al., 2011). The BADS-SF is a Likert scale consisting of nine 7-point items (from 0 = *Not at all* to 6 = *Completely*) arranged in two factors that are “*Activation*” (e.g., “*I was an active person and accomplished the goals I set out to do*”) and “*Avoidance*” (e.g., “*Most of what I did was to escape from or avoid something unpleasant*”). A total score, equal to the sum of all item scores, can be computed (items 1, 6, 7 and 8 are reverse scored). Two sub-scores corresponding to the two factors can also be calculated (items 1, 6, 7 and 8 are not reverse scored). The French version of the BADS-SF was used (Wagener et al., 2015).

#### Behavioural Inhibition System/Behavioural Activation System Scale (BIS/BAS Scale)

The BIS/BAS scale (Carver & White, 1994) assesses the behavioural inhibition system (BIS, e.g., “*I worry about making mistakes*”) and three facets of the behavioural activation system (BAS/Drive, e.g., “*If I see a chance to get something I want I move on it right away*”; BAS/Fun seeking, e.g., “*I’m always willing to try something new if I think it will be fun*”; BAS/Reward responsiveness, e.g. “*When I get something I want, I feel excited and energized*”). The BIS/BAS scale is a Likert scale composed of twenty-four 4-point items (from 1 = *Strongly agree* to 4 = *Strongly disagree*). All items are reverse scored except for items 2 and 22. A sum score is calculated for each subscale from the composing items. The French version of the BIS/BAS scale was used (Caci et al., 2007).

### Statistical analyses

The assessment of the structure of the French versions of the EROS and RPI was made by confirmatory factor analyses using LISREL software (Jöreskog & Sörbom, 2006). Item analyses, descriptive analyses and correlational analyses were also performed.

## Results

### Normative Analyses and Factor Structures

For the entire sample (119 men; 347 women), the mean item-total correlation for the ten items of the EROS was 0.46 (0.21–0.65) and for the 20 items of the RPI, this was 0.30 (0.00–0.65). The univariate normality of EROS and RPI’s data was examined by computing skewness and kurtosis of each item of each scale. The results highlighted that, for the EROS, skewness ranged from –0.61 to 0.47 and kurtosis from –0.83 to 0.61 and that, for the RPI, skewness varied from –1.28 to 0.36 and kurtosis from –0.95 to 1.45. These results did not indicate any strong deviation from normality as skewness’ absolute values were not greater than 3 and kurtosis’ were not above 20 (Weston & Gore, 2006). Multivariate normality was also examined by computing Mardia’s multivariate normality tests (Byrne, 2001; Mardia, 1974). These tests indicated that the data for the EROS (Mardia’s skewness = 6.78, p < 0.01; Mardia’s kurtosis = 138.20, p < 0.01) and for the RPI are not multivariate normal (Mardia’s skewness = 37.48, p < 0.01; Mardia’s kurtosis = 513.52, p < 0.01). Then, a method of estimation which can handle the lack of multivariate normality was employed to assess the factorial structure of the EROS and of the RPI: *Unweighted Least Squares* (ULS) (Blunch, 2008). Moreover, as Lei and Lomax (2005) indicated, goodness-of-fit indices which are computed on samples composed of at least 100 participants resist to the lack of multivariate normality.

Confirmatory factor analyses were conducted in order to assess the adequacy of the one-factor solution of the French version of the EROS and the two-factor solution of the French version of the RPI. Values of the goodness-of-fit indices are presented in Table 2. The one-factor solution for the EROS demonstrated good fit as RMSEA was equal to 0.06 (a value between 0.05 and 0.08 indicates a reasonable fit), CFI was 0.99 (a value superior to 0.97 indicates a good fit) and GFI was 0.99 (a value superior to 0.95 indicates a good fit) (Bentler, 1990; Schermelleh-Engel, Moosbrugger & Müller, 2003). The completely standardised factor loadings of the EROS items were all significant (Table 3) and greater than 0.30 (salient loading; Gorsuch, 1983). The two-factor solution for the RPI demonstrated a good fit as RMSEA was 0.03 and the CFI was 0.99. The GFI was equal to 0.84. The completely standardised factor loadings of the RPI were also all significant (Table 4) and greater than 0.30 (salient loading; Gorsuch, 1983). The composition of our sample does not allow to assess the factorial invariance according to sex, age and level of education. Indeed, each categories did not comprise enough participants to compute confirmatory factor analyses.

**Table 2:** Goodness-of-fit indices for the different models (*n* = 466).

Model	χ^2^	df	Normed χ^2^	RMSEA	GFI	AGFI	NNFI	CFI

**EROS : One-factor**	103.46	35	2.96	0.06	0.99	0.99	0.99	0.99
**RPI : Two-factor**	322.30	169	1.91	0.06	0.97	0.96	0.97	0.98

**Table 3:** Factor Loadings of French EROS items^2, 3^.

		Factor loading

1	A lot of activities in my life are pleasurable. *De nombreuses activités de ma vie sont plaisantes.*	0.83
2	I have found that many experiences make me unhappy^*^. J’ai pris conscience que de nombreuses expériences me rendent malheureux(se)^*^.	0.66
3	In general, I am very satisfied with the way I spend my time. *De manière générale, je suis satisfait(e) de la manière dont je passe mon temps.*	0.81
4	It is easy for me to find enjoyment in my life. *Il est facile pour moi d’éprouver du plaisir dans la vie.*	0.79
5	Other people seem to have more fulfilling lives^*^. Les autres semblent avoir des vies plus épanouies^*^.	0.75
6	Activities that used to be pleasurable no longer are gratifying^*^. Les activités qui étaient autrefois amusantes ne sont plus agréables^*^.	0.66
7	I wish that I could find more hobbies that would bring me a sense of pleasure^*^. J’aimerais trouver des hobbys qui me procurent un sentiment de plaisir^*^.	0.42
8	I am satisfied with my accomplishments. *Je suis satisfait(e) de mes réalisations.*	0.80
9	My life is boring^*^. Ma vie est ennuyeuse^*^.	0.83
10	The activities I engage in usually have positive consequences. *Les activités auxquelles je participe ont généralement des conséquences positives.*	0.71

^2^ Factorial weight stamped over 0.30. ^3^ Items followed by an “^*^” are reversed scores.

**Table 4:** Factor Loadings of French RPI items^4^.

		Factor 1 Probability of satisfaction	Factor 2 Environmental suppressors

1	I have many interests that bring me pleasure. *J’ai beaucoup de centres d’intérêt qui me procurent du plaisir.*	0.76	
2	I make the most of opportunities that are available to me. *J’exploite au maximum les opportunités qui se présentent à moi.*	0.65	
3	My behaviours often have negative consequences. *Mon comportement a souvent des conséquences négatives.*		0.72
4	I make friends easily. *Je me fais facilement des amis.*	0.56	
5	There are many activities that I find satisfying. *Il existe de nombreuses activités que je trouve satisfaisantes.*	0.82	
6	I consider myself to be a person with many skills. *Je me considère comme une personne dotée de nombreuses compétences.*	0.63	
7	Things happen that make me feel helpless or inadequate. *Il se passe des choses qui me font me sentir impuissant(e) ou inadapté(e).*		0.70
8	I feel a strong sense of achievement. *J’éprouve un fort sentiment d’accomplissement.*	0.79	
9	Changes have happened in my life that have made it hard to find enjoyment. *Dans ma vie se sont produits des changements qui m’empêchent d’éprouver du plaisir.*		0.80
10	It is easy to find good ways to spend my time. *Il est facile de trouver de bons passe-temps.*	0.66	
11	I have the abilities to obtain pleasure in my life. *J’ai la capacité à générer du plaisir dans ma vie.*	0.86	
12	I have few financial resources, which limits what I can do. *Mes ressources financières étant limitées, cela restreint mes activités.*		0.37
13	I have had many unpleasant experiences. *J’ai vécu de nombreuses expériences désagréables.*		0.68
14	It seems like bad things always happen to me. *On dirait que les malheurs n’arrivent qu’à moi.*		0.76
15	I have good social skills. *J’ai de bonnes aptitudes sociales.*	0.60	
16	I often get hurt by others. *Je me sens souvent blessé(e) par autrui.*		0.73
17	People have been mean or aggressive toward me. *Les gens ont été méchants ou agressifs à mon égard.*		0.68
18	I have been very capable in jobs I have had. *J’ai été très compétent(e) dans les postes que j’ai occupés.*	0.36	
19	I wish I could find a place to live that brought more satisfaction to my life. *J’aimerais trouver un lieu de vie qui m’apporte davantage de satisfaction dans la vie.*		0.53
20	I have many opportunities to socialize with people. *J’ai beaucoup d’opportunités de rencontrer des gens.*	0.63	

^4^ Factorial weight stamped over 0.30.

For the EROS, the one-factor solution accounted for 51.42% of the variance and for the RPI, the two-factor solution accounted for 91.97% (Factor 1: 48.14%; Factor 2: 43.83%). Factor 1 of the RPI was composed of items 1, 2, 4, 5, 6, 8, 10, 11, 15, 18, 20 and factor 2 of items 3, 7, 9, 12, 13, 14, 16, 17, 19. Because this two-factor structure was identical to the one reported in Carvalho, Gawrysiak et al. (2011), the factors for the French version are labelled as they are for the English version: factor 1 is “*Reward probability*”, assessing the probability of experiencing reinforcing events and the adequacy of instrumental behaviours, and factor 2 is “*Environmental suppressors*”, assessing the availability of environmental reinforcements and the risk of exposure to aversive and unpleasant experiences.

### Internal consistency and convergent validity

Internal consistency was assessed using Cronbach’s alphas. For the EROS, this was 0.89. Cronbach’s alpha for factor 1 of the RPI was 0.87 and 0.86 for factor 2. The Tables 5 and 6 present for each scale the values of Cronbach’s alpha if each item is deleted. Internal consistency for the other measures was mostly acceptable except for some subscales of the BIS/BAS scale. Descriptive statistics were performed on the whole sample (*n* = 466). Means, standard deviations and internal consistency coefficients of each scale (total and/or subscales scores) are presented in Table 7.

**Table 5:** Cronbach’s α values if items of the EROS are deleted.

	Cronbach’s α if deleted

ITEM 1	0.87
ITEM 2	0.88
ITEM 3	0.87
ITEM 4	0.87
ITEM 5	0.87
ITEM 6	0.88
ITEM 7	0.90
ITEM 8	0.87
ITEM 9	0.88
ITEM 10	0.88

**Table 6:** Cronbach’s α values if items of the RPI are deleted.

	Cronbach’s α if deleted

ITEM 1	0.88
ITEM 2	0.89
ITEM 3	0.89
ITEM 4	0.88
ITEM 5	0.88
ITEM 6	0.89
ITEM 7	0.89
ITEM 8	0.88
ITEM 9	0.88
ITEM 10	0.89
ITEM 11	0.88
ITEM 12	0.89
ITEM 13	0.89
ITEM 14	0.89
ITEM 15	0.89
ITEM 16	0.89
ITEM 17	0.89
ITEM 18	0.89
ITEM 19	0.89
ITEM 20	0.89

**Table 7:** Mean, standard deviation and Cronbach’s alpha of all instruments^5^ (*n* = 466).

		*M*	*SD*	Cronbach’s α

EROS		28.51	5.76	0.89
RPI	Factor 1	31.19	5.44	0.87
	Factor 2	21.10	5.18	0.86
BDI-II		13.00	11.36	0.94
BADS	Total	32.85	9.97	0.83
	Activation	13.32	5.53	0.86
	Avoidance	5.45	4.64	0.76
BIS/BAS SCALE	BIS	21.39	3.69	0.80
	BAS/Drive	8.90	2.11	0.63
	BAS/Fun seeking	10.73	2.18	0.63
	BAS/Reward responsiveness	16.15	2.07	0.55
STAI-Y Form B		45.95	9.81	0.88

^5^ EROS= Environmental Reward Observation Scale, RPI Factor 1= Probability of satisfaction, RPI Factor 2= Environmental suppressors, BDI-II= Beck Depression Inventory II, BADS= Behavioural activation for depression scale, BIS= Behavioural Inhibition System, BAS/Drive, BAS/Fun seeking, BAS/Reward responsiveness, STAI-Y Form B= State-Trait Anxiety Inventory-Y Form B

Convergent validity was examined by computing (Pearson’s correlations) relations between the EROS, the RPI and the other measurements (Table 8). First of all, EROS and factor 1 of the RPI were highly correlated with each other, which supports the convergent validity of these two measures. Also supporting the convergent validity of these measures, moderate-to-strong positive correlations were observed between both the EROS and the RPI and the BADS-SF (total and activation score) and two of the BAS scales (fun seeking and reward responsiveness). The convergent validity was also supported by moderate-to-strong positive correlations between factor 2 of the RPI and the BDI-II, STAI-B, BADS-SF (avoidance) and BIS. Finally, the moderate-to-strong negative correlations observed between both the EROS and the RPI and the BDI-II, the STAI-B and the BADS-SF (avoidance) and the BIS scale also indicate the convergent validity of the EROS and the RPI as well as the moderate-to-strong negative correlations between factor 2 of the RPI and the BADS-SF total and activation scores.

**Table 8:** Pearson’s correlations between the self-report measures (*n* = 466)^6^.

	1	2a	2b	3	4	5	5a	5b	6	7	8	9

EROS	–	0.75^**^	–0.71^**^	–0.75^**^	–0.76^**^	0.71^**^	0.62^**^	–0.51^**^	–0.45^**^	–0.01	0.17^**^	0.14^**^
2a. RPI – Factor 1		–	–0.50^**^	–0.59^**^	–0.63^**^	0.60^**^	0.59^**^	–0.34^**^	–0.40^**^	0.06	0.32^**^	0.22^**^
2b. RPI – Factor 2			–	0.72^**^	0.74^**^	–0.62^**^	–0.45^**^	0.57^**^	0.50^**^	0.08	0.00	0.05
BDI-II				–	0.75^**^	–0.70^**^	–0.56^**^	0.57^**^	0.43^**^	0.04	–0.06	–0.05
STAI-B					–	–0.71^**^	–0.55^**^	0.62^**^	0.66^**^	0.03	–0.12^**^	–0.01
BADS-SF						–	0.87^**^	–0.74^**^	–0.41^**^	–0.06	0.08	0.07
5a. BADS-Activation							–	–0.37^**^	–0.28^**^	0.00	0.12^*^	0.12^**^
5b. BADS-Avoidance								–	0.40^**^	0.12^**^	–0.00	0.05
BIS									–	0.03	–0.06	0.26^**^
BAS/Drive										–	0.31^**^	0.33^**^
BAS/Fun seeking											–	0.41^**^
BAS/Reward responsiveness												–

^6^^*^ p < 0.05, ^**^ p < 0.001

## Discussion

The aim of our study was to validate the French versions of the Environmental Reward Observation Scale and the Reward Probability Index, and to present the psychometric properties of these translated versions. The validation of these scales aimed to provide French-speaking clinicians and researchers with new and sound assessment tools. Furthermore, the brevity of these two scales makes them quite practical and accessible: completion of both scales only takes between five to ten minutes. Overall, the results are very satisfying. Indeed, findings from the present study reveal that the French versions of both the EROS and the RPI possess adequate psychometric properties.

Concerning the EROS, confirmatory factor analysis indicates that a one-factor solution yields a good fit. These results corroborate those reported in Armento and Hopko (2007). Thus, combined with convergent measures such as the RPI, the sum of all items in the EROS gives a score of environmental rewards, with a higher score indicating higher environmental rewards.

Regarding the RPI, based on confirmatory factor analysis, a two-factor solution yields good fit parameters. The factor “*Reward probability*” (factor 1) assesses the probability of experiencing reinforcing events and the adequacy of instrumental behaviours while the “*Environmental suppressors*” factor (factor 2) evaluates the availability of environmental reinforcements and the risk of exposure to aversive and unpleasant experiences. Consequently, the French version of the RPI allows one to calculate two different scores, depending on the aim of the clinical application or on the objective of the research.

The psychometric properties of these two translated scales are very satisfying. Indeed, our results revealed high internal consistency of the EROS and of the two RPI subscales. Moreover, the convergent validity of these two scales was confirmed as they strongly correlated with measures of depression, anxiety, activation, avoidance and behavioural systems (inhibition and activation). High environmental rewards appear to be closely related to activation and the behavioural activation system, as Armento and Hopko (2007) evocated. On the contrary, low environmental rewards appear to be closely related to depression, anxiety, avoidance and behavioural inhibition. These findings are consistent with those of previous research: being exposed to rewarding activities or events is related to lower levels of self-reported depression (Armento & Hopko, 2007; Carvalho, Gawrysiak et al., 2011; Lewinsohn et al., 1980). This finding confirmed the postulates of the behavioural theory of depression (Lewinsohn, 1974).

The results of this study and of previous ones (Armento & Hopko, 2007; Carvalho, Gawrysiak et al., 2011) suggest that the approximation of response-contingent positive reinforcements relies on several facets. Therefore, in the current framework of behavioural models of depression, the use of both the EROS and the RPI in clinical practice as well as in research should permit new insights in the mechanisms that underlie depression. Moreover, the combined utilisation of the EROS and the RPI also seems relevant because these scales assess the perception of environmental rewards, while changing this perception can be one of the main aims of psychotherapy. The utilisation of both the EROS and the RPI can also be quite useful when combined with daily activities diaries as recommended by Ryba and Hopko (2012). This combination might help to examine if an objective increase of experienced activities is paralleled by an increase in EROS and RPI (factor 1) scores. We also recommend using the EROS and RPI simultaneously with the BADS-SF while we must pay close attention to the difference of assessment period: the EROS and RPI assess the last months while the BADS-SF assesses the last week. This combination will respond to the behavioural activation model’s requirement since both functional behaviour and contact with environmental reinforcement are measured. Moreover, it seems to be of interest to conjointly use the EROS and the BIS/BAS scale. The former scale assesses the frequency of contact with positive reinforcement while the latter scale, and more specifically the BAS subscales, assess the emotional consequences of experiencing reward, the motivation to pursue reward and the desire for enjoyment. Thus, the combined utilisation of the above-mentioned scales can help the clinician and researcher, as well as the client, to have a broader overview of the client’s psychological state. Finally, it is noteworthy that our results confirmed that avoidance and environmental rewards are negatively related. Avoidance is known to negatively influence mood, which is why decreasing avoidance is usually a main aim in psychotherapy (Martell et al., 2001). In our opinion, this finding again underlines the importance of working on the environmental contingencies that lead, or do not lead, to rewards.

This study presents some limitations and provides guidelines for future research. First, the sample was relatively young (*M* = 36.5) and had a high level of education. Therefore, it remains to be shown that these results are generalisable across different age groups and levels of education. Also, the sample consisted mostly of women (74.46%). Since the rationale of both the EROS and the RPI is based on empirically sound constructs and whose reliability has been previously demonstrated in both women and in men (Lewinsohn, 1974), we believe that the disproportionate gender representation does not influence the factorial structure of these two scales. Nonetheless, according to the results of a recent study by Ryba and Hopko (2012), we believe that gender might influence the scores obtained on each scale. Indeed, they demonstrated that gender differences exist; in particular, they found that women have higher scores on behavioural events and engage in more behavioural domains, compared to men. This observation is also strengthened by the notion that women seem to be more sensitive to reinforcements and rewards (Tull, Gratz, Latzman, Kimbrel & Lejuez, 2010). Thus, research aiming to establish norms for the EROS and RPI will probably reveal differences between women and men. Because women suffer more often of depression than men and give a higher average value to behavioural events, Ryba and Hopko (2012) explored the effect of the environmental reward as a moderator of gender and depression. These results showed that in this case, gender and depression are less related. These results seem to indicate the importance of the psychotherapeutic work on environmental reward, especially with women. Because our sample did not allow the possibility to examine the factorial invariance according to sex, age and level of education, the utilisation of the EROS and the RPI in samples which do not correspond to this one should be made with caution. Second, the participants were not recruited in clinical settings, however, the link between low mood and decrease in response-contingent positive reinforcement is mostly obvious in that kind of setting (Hopko et al., 2003). Therefore, it might be interesting to assess the factorial structure of both the EROS and the RPI in such clinical samples. This approach might provide more information on their discriminant validities. Notwithstanding, it is noteworthy that the present sample presents a mean depression score indicating the presence of mild depression (BDI-II: *M* = 13.00, *SD* = 11.36). As a consequence, this sample cannot be considered as non-experiencing depressive symptoms at all. Furthermore, according to Borsboom, Cramer, Schmittmann, Epskamp and Waldrop (2011) and Kinderman (2009), psychological difficulties are spread on a continuum from non-clinical to clinical ones. Thus, the absence of clinical participants does not seem, in our opinion, to limit generalisability of our findings much. The lack of clinical participants in the assessment of the factorial structures of self-reported measures mainly consists in a limitation for important psychological disorders such as schizophrenia or neurological disorders because these disorders affect the consciousness. Nevertheless, it might be interesting to assess the factorial structure of both the EROS and the RPI in clinical samples. Thirdly, discriminant validity of the EROS and the RPI should be investigated in relation to other assessment tools that focus on concepts which are related to environmental rewards, but remain different. The Multidimensional Scale of Perceived Social Support (Zimet, Dahlem, Zimet & Farley, 1988) might be one such scale, as social support can be conceptualised as some other kind of environmental reward. Fourthly, it might be useful to assess reliability and validity of these scales with additional statistical techniques such as test-retest reliability and/or predictive validity. It might be interesting for future studies to address these issues.

## Conclusion

The French versions of both the EROS and the RPI appear to be reliable and valid assessments of environmental rewards and reliable proxy measures of response-contingent positive reinforcement. Therefore, it is advised both instruments can be used for screening in research and clinical practice. Moreover, it would be of interest if the EROS and the RPI were translated into additional languages other than English and French.

## References

[1] Armento M., Hopko D. (2007). The Environmental Reward Observation Scale (EROS): Development, validity, and reliability. Behavior Therapy.

[2] Beck A., Steer R., Brown G. (1996). Manual for the Beck Depression Inventory-II.

[3] Bentler P. (1990). Comparative fit indexes in structural models. Psychological Bulletin.

[4] Blunch N. (2008). Introduction to structural equation modelling using SPSS and AMOS.

[5] Borsboom D., Cramer A., Schmittmann V., Epskamp S., Waldorp L. (2011). The Small World of Psychopathology. PLos ONE.

[6] Byrne B. (2001). Structural equation modeling with AMOS: Basic concepts, applications, and programming.

[7] Caci H., Deschaux O., Baylé F. (2007). Psychometric properties of the French versions of the BIS/BAS scales and the SPSRQ. Personality and Individual Differences.

[8] Carvalho J., Gawrysiak M., Hellmuth J., McNulty J., Magidson J., Lejuez C., Hopko D. (2011). The Reward Probability Index: Design and validation of a scale measuring access to environmental reward. Behavior Therapy.

[9] Carvalho J., Hopko D. (2011). Behavioral theory of depression: Reinforcement as a mediating variable between avoidance and depression. Journal of Behavior Therapy and Experimental Psychiatry.

[10] Carvalho J., Trent L., Hopko D. (2011). The impact of decreased environmental reward in predicting depression severity: Support for behavioral theories of depression. Psychopathology.

[11] Carver C., White T. (1994). Behavioral inhibition, behavioral activation, and affective responses to impending reward and punishment: The BIS/BAS scales. Journal of Personality and Social Psychology.

[12] Centre de psychologie appliquée (1996). Manuel du BDI-II.

[13] Correia C., Carey K., Borsari B. (2002). Measuring substance-free and substance related reinforcement in the natural environment. Psychology of Addictive Behaviors.

[14] Cuijpers P., van Straten A., Warmerdam L. (2007). Behavioral activation treatments of depression: A meta-analysis. Clinical Psychology Review.

[15] Gauthier J., Bouchard S. (1993). Adaptation canadienne-française de la forme révisée du State Trait Anxiety Inventory de Spielberger. Revue Canadienne des Sciences du Comportement.

[16] Gorsuch R. (1983). Factor analysis.

[17] Hopko D., Armento M., Cantu M., Chambers L., Lejuez C. (2003). The use of daily diaries to assess the relations among mood state, overt behavior, and reward value of activities. Behaviour Research and Therapy.

[18] Hopko D., Lejuez C., Ruggiero K., Eifert G. (2003). Contemporary behavioral activation treatments for depression: Procedures, principles, and progress. Clinical Psychology Review.

[19] Jöreskog K., Sörbom D. (2006). LISREL 8.80 for Windows [computer software].

[20] Kanter J., Busch A., Rusch L. (2009). Behavioral Activation.

[21] Kinderman P. (2009). Understanding and Addressing Psychological and Social Problems: The Mediating Psychological Processes Model. International Journal of Social Psychiatry.

[22] Lei M., Lomax R. (2005). The effect of varying degrees of nonnormality in structural equation modeling. Structural equation modeling.

[23] Lejuez C., Hopko D., Acierno R., Daughters S., Pagoto S. (2011). Ten Year Revision of the Brief Behavioral Activation Treatment for Depression (BATD): Revised Treatment Manual (BATD-R). Behavior Modification.

[24] Lejuez C., Hopko D., Hopko S. (2001). A Brief Behavioral Activation Treatment for Depression. Behavior Modification.

[25] Lewinsohn P., Friedman R., Katz M. (1974). A behavioral approach to depression. The psychology of depression: Contemporary theory and research.

[26] Lewinsohn P., Sullivan J., Grosscup S. (1980). Changing reinforcing events: An approach to the treatment of depression. Psychotherapy: Theory, Research and Practice.

[27] MacPhillamy D., Lewinsohn P. (1974). Depression as a function of levels of desired and obtained pleasure. Journal of Abnormal Psychology.

[28] Manos R., Kanter J., Busch A. (2010). A critical review of assessment strategies to measure the behavioral activation model of depression. Clinical Psychology Review.

[29] Manos R., Kanter J., Luo W. (2011). The behavioral activation scale for depression-short form: Development and validation. Behavior Therapy.

[30] Mardia K. (1974). Applications of some measures of multivariate skewness and kurtosis in testing normality and robustness studies. Sankhya Series B.

[31] Martell C., Addis M., Jacobson N. (2001). Depression in Context: Strategies for Guided Action.

[32] Martell C., Dimidjian S., Herman-Dunn R. (2013). Behavioral activation for depression: A clinician guide.

[33] Mazzucchelli T., Kane R., Rees C. (2010). Behavioral activation interventions for well-being: A meta-analysis. Journal of Positive Psychology.

[34] Ryba M., Hopko D. (2012). Gender Differences in Depression: Assessing Mediational Effects of Overt Behaviors and Environmental Reward through Daily Diary Monitoring. Depression Research and Treatment.

[35] Schermelleh-Engel K., Moosbrugger H., Müller H. (2003). Evaluating the fit of structural equation models: Test of significance and descriptive goodness-of-fit measures. Methods of Psychological Research.

[36] Spielberger C., Gorsuch R., Lushene R., Vagg P., Jacobs G. (1983). Manual for the State-Trait Anxiety Inventory.

[37] Tavakol M., Dennick R. (2011). Making sense of Cronbach’s alpha. International Journal of Medical Education.

[38] Tull M., Gratz K., Latzman R., Kimbrel N., Lejuez C. (2010). Reinforcement sensitivity theory and emotion regulation difficulties: A multimodal investigation. Personality and Individual Differences.

[39] Wagener A., Van der Linden M., Blairy S. (2015). Psychometric properties of the French translation of the Behavioral Activation for Depression Scale – Short Form (BADS-SF) in non-clinical adults. Comprehensive Psychiatry.

[40] Weston R., Gore P. (2006). A brief guide to structural equation modeling. The Counseling Psychologist.

[41] White N. (1989). Reward or reinforcement: What’s the difference?. Neuroscience and Biobehavioral Review.

[42] Zimet G., Dahlem N., Zimet S., Farley G. (1988). The Multidimensional Scale of Perceived Social Support. Journal of Personality Assessment.

